# Online Aerial Terrain Mapping for Ground Robot Navigation

**DOI:** 10.3390/s18020630

**Published:** 2018-02-20

**Authors:** John Peterson, Haseeb Chaudhry, Karim Abdelatty, John Bird, Kevin Kochersberger

**Affiliations:** Virginia Tech Unmanned Systems Laboratory, Blacksburg, VA 24060, USA; haseeb7@vt.edu (H.C.); karim@vt.edu (K.A.); jobird@vt.edu (J.B.); kbk@vt.edu (K.K.)

**Keywords:** unmanned aircraft, air–ground cooperation, multi-robot coordination

## Abstract

This work presents a collaborative unmanned aerial and ground vehicle system which utilizes the aerial vehicle’s overhead view to inform the ground vehicle’s path planning in real time. The aerial vehicle acquires imagery which is assembled into a orthomosaic and then classified. These terrain classes are used to estimate relative navigation costs for the ground vehicle so energy-efficient paths may be generated and then executed. The two vehicles are registered in a common coordinate frame using a real-time kinematic global positioning system (RTK GPS) and all image processing is performed onboard the unmanned aerial vehicle, which minimizes the data exchanged between the vehicles. This paper describes the architecture of the system and quantifies the registration errors between the vehicles.

## 1. Introduction

This work presents a multi-robot system for efficient exploration and navigation in outdoor environments. The system consists of a ground vehicle (the Clearpath Jackal) , and a custom multi-rotor. The objective of the mission is to quickly and efficiently drive an unmanned ground vehicle through an unknown outdoor environment. Ground vehicles are typically equipped with a variety of sensors for navigation and obstacle avoidance. Ultrasonic, Light Detection and Ranging (LIDAR), camera, and radar sensors are just a few potential examples. These sensors have different maximum ranges, but they share the requirement for line of sight for obstacle detection, ignoring radar reflections. Many algorithms for efficient path planning exist. Common algorithms, including A*, will plan optimally in discrete environments and there are extensions to continuous coordinates. However, the optimality of these paths is limited by the planner’s knowledge of obstacles. The line of sight of sensors on a ground vehicle is limited by the geometry of the environment. In indoor planar environments, line of sight is limited by walls and other obstacles, but in an outdoor environment the ground surface and vegetation also restrict it. For outdoor robots, a common way to improve the range of line of sight is to mount the sensor higher up on the vehicle. This provides the vehicle with a better perspective to see over hills and tall grass.

A logical extension is to use a remote sensor mounted on an aerial vehicle to obtain a completely different perspective on the scene. When using an overhead downwards-facing perspective, there is a compromise between area covered and resolution on the ground. At the extreme, a satellite in orbit can see large portions of the Earth’s surface at any given moment, but there are restrictions on the clarity of these images because of the atmosphere and limitations on the time of day that data may be acquired, as dictated by the satellite’s orbit. This data may be too out of date to be useful for navigation in dynamic environments. Fixed wing aircraft can circle an area for extended periods of time, but their size and airspeed may require observation to be done from a higher altitude than is ideal. A common way to increase the spatial resolution from the air is to use higher-resolution cameras. However, in their absence, another approach is to fly at lower altitudes, acquire multiple images of subsets of the mission area, and combine the images into a single high-resolution map.

This system uses a multi-rotor to capture aerial imagery of a mission area to construct a terrain map to efficiently navigate a ground robot to a destination. Planners typically minimize path length which does not necessarily minimize energy consumption. In radiation search operations using scene understanding with autonomous unmanned aerial vehicles (UAVs) and unmanned ground vehicles (UGVs) in [1], the authors show the difference in the power consumed by a ground vehicle traversing grass and pavement. This work utilizes overhead imagery to derive terrain classes, infers traversibility costs from these terrain classes, generates an energy-efficient trajectory through this cost map, and then commands the ground vehicle to follow this trajectory.

## 2. Background

It is common to use aerial maps to augment navigation by ground vehicles. Before its use in robotics, there was clearly a benefit to using a higher vantage point, which afforded a greater and more unobstructed field of view. Within robotics, several different approaches are taken to leverage aerial data for ground robot navigation. Many approaches consider the aerial data collection as a separate mission that might occur days or weeks before the ground vehicle is deployed on the scene. This implies some amount of human intervention in data processing, such as moving files from one vehicle to another, or manually running software on the acquired data. In these surveying missions, an entire area might be imaged or scanned in advance to ensure that aerial data is available for the entire region. Unlike some of these previous works, the approach presented in this paper aims to tightly couple the aerial data collection with the ground level navigation. Rather than blindly scanning an entire area, the aerial vehicle only surveys the area required for the ground vehicle to reach its goal. Given that this work covers aerial mapping, multi-robot systems, computer vision, and ground-level navigation, there are several works which overlap in part or in whole with the system presented here.

In [2], Templeton et al. presented a vision-based terrain mapping system for landing an autonomous helicopter. Their system fuses feature tracking with a global positioning system (GPS) and inertial measurement system (INS) to estimate the global position and orientation of the camera. This estimate of the motion of the camera is used to estimate the 3D position of tracked points. In this case, no ground vehicle is involved at all, but their terrain mapping procedure is online to enable the helicopter to make its autonomous landing.

In [3], Papadakis presented a relatively recent survey covering visual and LIDAR-based terrain traversability estimation methods. The method employed in this paper is meant to be a place holder until a more sophisticated algorithm, perhaps from the set presented in Papadakis’ work can be selected and used.

One of the components of this system is an operator control station (OCS) that enables the operator to effectively supervise and control the two vehicles. In [4] Owens et al. created an OCS to enable a single operator to control a large team of robots. Their OCS constructs a visualization of the environment by applying aerial imagery as a texture to a mesh of the environment in real time. However, this mesh is constructed from pre-collected LIDAR scans of the area with 1-m resolution. Their texturing approach is actually quite similar to the method employed in this paper for image assembly.

Quite a few methods take the approach of collecting and preprocessing the aerial data so the ground vehicle is just given a path and follows it. In one of the earlier works in this specific area [5], Stentz et al. treated the aerial perspective as being just a mobile sensor of the ground vehicle. They demonstrated that the aerial perspective of the ground improved the efficiency of planning, even when the maps generated were stale due to changes in the environment. However they were unable to autonomously fly the UAV, so they relied on manual flight of the UAV to collect data from the area before deploying the ground vehicle. The authors did conduct some simulation work to explore the concept of UAV exploration. In a work the following year by Stentz et al. [6], the authors improved the registration of aerial collected laser scans and improved the stereo algorithm and hardware.

In [7] Vandapel et al. created a terrain mapping system utilizing aerial collected LIDAR scans. Their method, like other LIDAR-based methods, separates vegetation from the ground surface to generate a traversability map. This particular paper focused on evaluating the quality of the generated terrain maps. In a subsequent work [8], Vandapel et al. went into greater detail, describing their multi-robot system that augments the ground navigation of a ground vehicle with pre-collected aerial LIDAR data. In this work, they register a mesh generated from ground-level laser scans with the mesh generated from aerial collected laser scans to localize the ground robot in the prebuilt map, rather than relying on the GPS.

Similarly, the authors of [8,9] examined the registration of LIDAR between aerial and ground vehicles by adapting the iterative closest point (ICP).

In [10] MacArthur et al. used a stereo system on a UAV to identify simulated land mines in a images, localize marked points in the images of an open field, and send a UGV to those locations. In [11] the authors used a combination of satellite-based imagery and data from the Shuttle Radar Topography Mission to generate an aerial terrain map. They planned a risk-sensitive path using A* on this terrain map for a ground vehicle. Naturally all of this space based overhead data was collected long in advance of the ground vehicle’s mission.

Chaimowicz et al. in [12] deployed a large multi-robot system consisting of both aerial and ground vehicles. They explored two methods of localization for their aerial vehicles, one using the GPS and INS of the UAV for its localization, and the other method based on image features correspondences. They demonstrated their localization and navigation systems and conducted an experiment where they localized one of the ground vehicles from the air. They ultimately found that both the GPS/INS method and the image feature-based methods had their limitations and neither method was perfect.

In [13], Hudjakov and Tamre applied neural network-based cost generation to pre-constructed orthophotos of the mission area. The authors use negative weights for the output of the neural network to represent easily traversable classes such as road and grass, with positive weights assigned to impassable terrains. They then applied a positive offset to the output for test data to ensure that all costs were positive for path planning with A*. The resulting path however does not appear to have been executed on a ground vehicle.

Giakoumidis et al. presented a more recent take on a small-scale indoor implementation of a multi-robot collaborative UAV–UGV system in [14]. They employed a Hue Saturation Value (HSV) classifier for obstacle detection in an indoor environment on imagery stitched together using Scale-Invariant Feature Transform (SIFT) features. Finally, they generated a slowness map which represents a distance transform to the nearest obstacle at each point in the map, and planned a minimum time trajectory for a ground robot.

Over the past 15 years, a variety of work has been done in autonomous collaborative UAV and UGV systems, but there has been relatively little research specifically on methods that operate both vehicles simultaneously and process data online.

## 3. Problem Statement

The goal of this project is to construct a multi-robot system to efficiently navigate an environment with a ground robot. Ground robots normally navigate using data collected from onboard sensors, however these sensor have limited range and field of view, and are subject to line of sight restrictions. Because of these limitations, navigation in partially explored environments requires replanning as obstacles and impassible terrain are discovered. Even if the underlying planning algorithm used by the ground vehicle is capable of generating efficient paths, the actual path taken by the vehicle will not be the shortest possible path because it will have to explore and replan. The only way to ensure that the UGV will plan and navigate optimally is to have complete knowledge of the mission area. By using a UAV to scout ahead without being hindered by terrain, we can gather this data efficiently and plan the path of the ground vehicle with more complete information.

Unlike some of other UAV–UGV collaborative systems, this one has the goal of operating in real time, simultaneously. While the UAV is still flying in the air collecting imagery, the goal is to be able assemble that imagery into an aerial terrain map, plan a path for the ground vehicle, and successfully navigate the ground vehicle to the destination, so that the whole process may be repeated for the same flight.

## 4. Assumptions

To construct this system in a timely fashion, several simplifying assumptions were made. In principle, these assumptions could be relaxed without changing the architecture of the system, but it would require the substitution of several components in the software. The greatest assumption that this system makes is that the environment is approximately planar. In terms of hardware, a real-time kinematic global positioning system (RTK GPS) base station defines the origin of the common coordinate frame between the robots. The height of this antenna above the ground is measured by hand and used to define the height of the ground plane in the common coordinate frame. This assumption of an approximately 2D ground plane simplifies the obstacle mapping of each vehicle and navigation tasks of the ground vehicle.

For the ground vehicle, this allows the open source package Move Base [15] to be used with a single plane-scanning LIDAR. Move Base operates on 2D obstacle maps, so future 3D reconstructions such as height maps or occupancy voxel grids which can be converted into a 2D obstacle map will still function.

On the aerial vehicle, this simplifies the computer vision task for assembling an orthomosaic and allows the use of a single monocular camera. For the sake of simplifying the obstacle detection with a monocular camera, we assume that obstacles that the UGV is unable to pass through are denoted with a single, known, solid color. In the future, the UAV will be equipped with a stereo camera pair to construct height maps in addition to the colored orthomosaic maps. This will allow obstacles to be naturally detected as discontinuities in the height map.

We also assume that the mission area has been defined in advance and is relatively small, on the order of half a square kilometer. This reduces the requirements in the range of the radios employed and enables modest fixed-sized maps to be used by the software, rather than more sophisticated scrolling implementations.

## 5. Approach

The system presented here consists of a pair of robots which are supervised and directed from an OCS. The Robot Operating System (ROS) is used to integrate open source and custom software components together providing a framework for organizing the software and interfacing with the hardware.

### 5.1. Hardware

**Figure 1:**
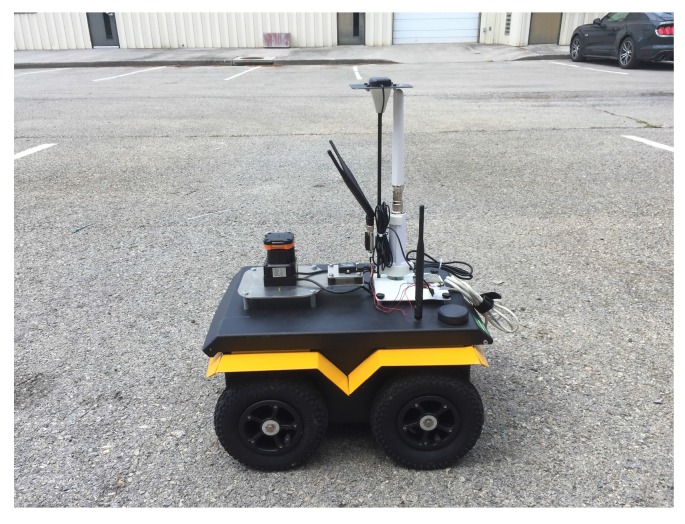
The Jackal unmanned ground vehicle shown with the first-generation Swifnav Piksi real-time kinematic global positioning system (RTK GPS) unit.

**Figure 2:**
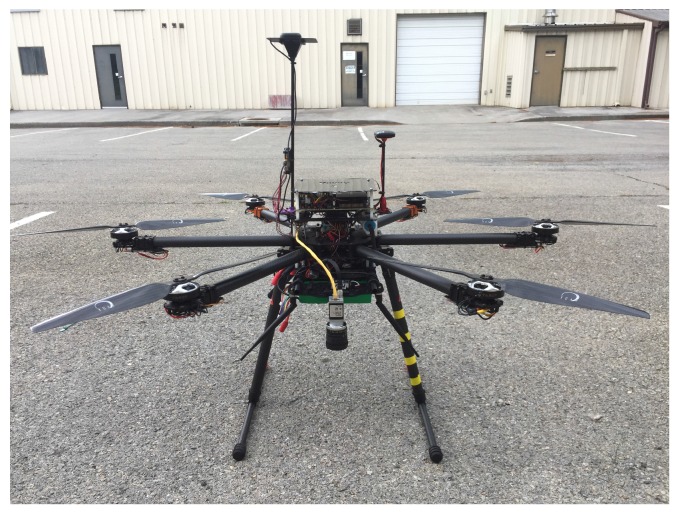
A custom unmanned aerial vehicle shown with the first-generation Swifnav Piksi RTK GPS unit.

The system consists of two mobile robots, a Jackal (a small ground robot produced by Clearpath Robotics [16] shown in Figure 1), and a custom hexacopter named the Bogey (shown in Figure 2).

The multi-rotor is a hexacopter equipped with a Pixhawk flight controller running Arducopter firmware [17] and an Nvidia TX2 computer [18]. The Pixhawk flight controller is responsible for the low-level stability of the aircraft, provides a position interface for moving the aircaft, and performs state estimation fusing an inertial measurement unit (IMU), magnetometer, GPS, and barometric pressure into an extended Kalman filter (EKF). MAVROS [19] implements the MAVLink communication protocol enabling the ROS system to interface with the flight controller to receive state estimates and sensor data as well as issue commands. The UAV is equipped with a Basler acA1920–155uc for imaging.

A low-level controller on the Jackal combined with a ROS driver node from Clearpath Robotics offers a ROS-based interface for the Jackal hardware out of the box. The Jackal is equipped with a Hokuyo UTM–30LX mounted parallel to the ground for obstacle detection. The Jackal’s integrated magnetometer and IMU are combined with wheel odometry for orientation estimation, while GPS position fixes are combined with wheel odometry for position estimation in an EKF.

Both vehicles are equipped with 5.8 GHz Ubiquiti Bullet M5 radios which both connect to a Rocket M5 radio at the ground station. These radios provide shell access to the onboard computers of the robots for launching and monitoring software at a low level and are used to relay data and commands from the OCS. These radios provide approximately 100 Mbps of bandwidth split between the two connections.

To establish a common coordinate frame between the vehicles, both are equipped with Piksi RTK GPSs. The RTK GPS measures position relative to a fixed base station with more precision than un-augmented GPSs, but it requires a real-time link from the base station to each rover unit for correction signals. This system uses a dedicated telemetry radio at the base station, and one on each vehicle, solely for these corrections.

**Figure 3:**
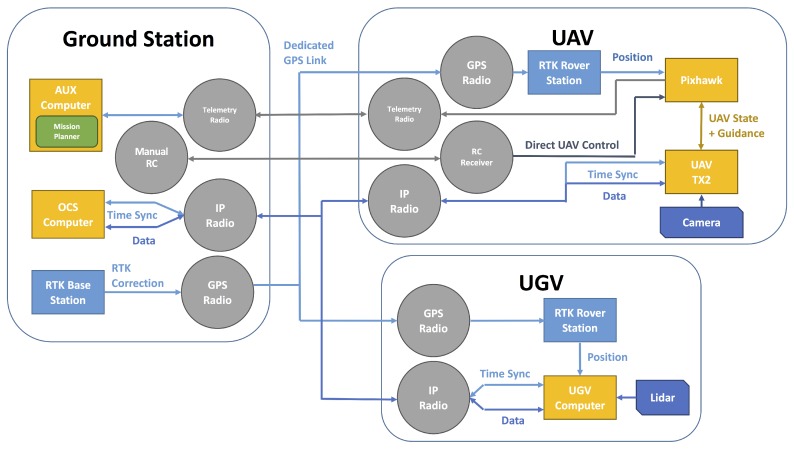
Diagram displaying the wireless links and sensor layout. OCS: operator control station; UAV: unmanned aerial vehicle; UGV: unmanned ground vehicle; IP: Internet protocol.

The UAV has two additional radio links. A telemetry link to a separate computer running Mission Planner [20], enables the pilot in command to monitor the flight controller’s state independently of the higher-level ROS-based software system. The other link is a connection to a Radio Control (RC) for the pilot to control the UAV manually during take offs, landings, and to override the autonomous system. Figure 3 displays these radio links and high-level components.

### 5.2. System Architecture

The multi-robot system utilizes a ROS to integrate a variety of open source packages. The ROS was chosen because it is a form of well supported robotics middleware which enables the construction of complex systems from individual components, many of which are already widely available. The ROS provides a publisher subscriber architecture for passing data between processes, called nodes, within a single computer and between computers. Service and Action interfaces enable synchronous execution of actions by other processes and provide critical feedback on the state of the execution. Launch files can be written to automate the start up of complex systems.

### 5.3. Mission and Algorithm

There are four computers in the multi-robot system: two at the base station and one onboard each vehicle. The computer running Mission Planner interfaces directly with the flight controller and is independent of the rest of the software.

**Figure 4:**
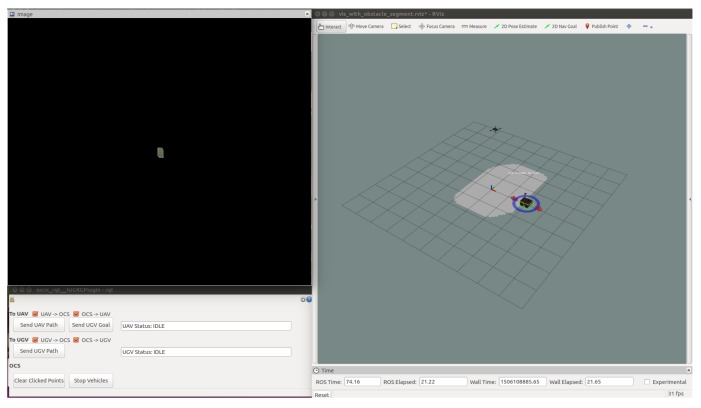
A sample image of the operator control station (OCS) presented to the user. The right hand window is an RViz window displaying the robots, cost maps, and paths. The image window in the upper left-hand corner visualizes the assembled image of the ground plane. The control widget in the lower left-hand corner displays the current status of the vehicles and their comms bridges. This interface allows goals to be specified and planned paths to be visualized before execution.

The OCS provides a visualization that displays the state of both of the robots as well as an interface for the operator to control the two vehicles. The interface, shown in Figure 4, consists of an RViz window which displays 3D models of the two vehicles depicting relative position of the two vehicles in the common coordinate frame with both the obstacle map constructed by the ground vehicle and synchronized with the UAV, and the terrain-based cost map constructed by the UAV. The state of the vehicles, planning, and moving, as well as error conditions, are also displayed to the operator.

**Figure 5:**
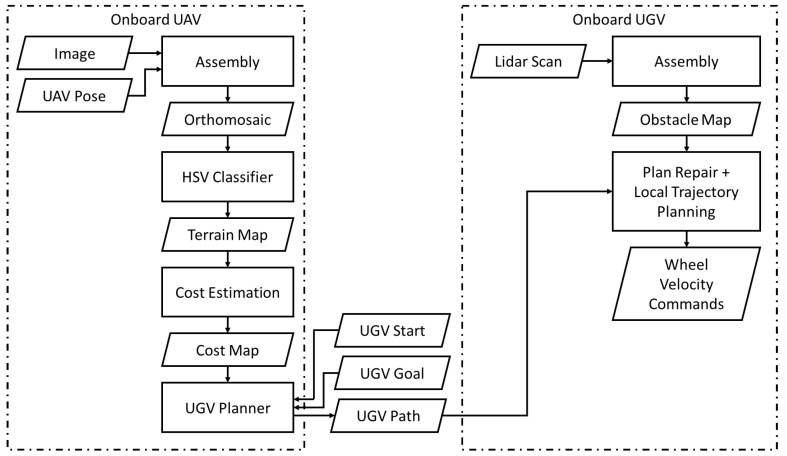
Flow chart displaying the mapping, path planning, and path execution pipelines running onboard the UAVs and UGVs. HSV: Hue Saturation Value.

The OCS provides an interface offering several different levels of control of the robots. At the lowest level, the interface enables direct teleoperation the ground vehicle using either a XBox controller or interactive markers. At an intermediate level, a point and click interface for each vehicle allows goals to be specified for each vehicle and invokes the onboard of each planner to autonomously navigate the vehicles to the specified locations. At the highest level, the mission interface enables the operator to execute the full mission of navigation by the UAV, planning for the ground vehicle, then navigation by the ground vehicle. Figure 5 displays an outline of the processes of planning the ground vehicle’s path, first onboard the UAV, then on transmitting it to the UGV for execution.

As a stand-in for more sophisticated exploration algorithms, the operator specifies a sequence of waypoints to control the area covered by the UAV with imagery. Each point specifies the (x,y) coordinate of the waypoints while a slider specifies the altitude of the UAV in the common coordinate frame. The user also specifies a corridor width parameter. The sequence of waypoints, starting from the initial position of the vehicle, defines a sequence of line segments, and these are expanded into rectangular regions by half the width on each side. Camera calibration was performed using the AprilCal [21] calibration toolbox which provides an estimate of the field of view of the camera. The UAV calculates the area covered by a single image on the ground plane using the camera intrinsics and specified altitude, and then assuming continuous capture, computes the amplitude and frequency of a constant altitude sine wave trajectory through the air to image each corridor in the sequence.

The UAV utilizes *MoveIt!* [22] for its trajectory planning and execution. By imagining a four-degree of freedom (4DOF) arm consisting of three perpendicular prismatic joints, ending with a yaw joint, and considering the UAV itself as the end effector, *MoveIt!* can be used to plan and execute the motions of the UAV. The *x* prismatic joint points east, the *y* prismatic joint points north, and the *z* prismatic joint points up. These joints are rooted at the arming position of the UAV. The frame MAVROS accepts commands in, allowing the commanded joint values to be converted directly into position and yaw set-points which the flight controller attempts to achieve. *MoveIt!* is configured to apply velocity and acceleration limits to each of these joints which are conservative estimates of the true capabilities of the vehicle, so the generated time stamped sequence of position and orientation commands for the vehicle is feasible. The flight controller’s top priority always remains the stability of the aircraft. In the event that a set-point position is too far away from the vehicle to be achieved in planned amount of time, the vehicle would simply move to the goal at best possible speed and the movement would halted if the vehicle did not reached the goal within the time limit. This allows the system to leverage the planning, control, trajectory execution, and monitoring capabilities that *MoveIt!* already implements. This also provides the capability for performing obstacle avoidance with the UAV if a source of 3D data becomes available in the future. However, this does introduce the additional complexity of converting the state published by MAVROS into joint states of this imagined 4DOF arm, as well as converting the joint state commands output by *MoveIt!* back into set-point position commands for MAVROS to send to the flight controller.

The UAV continuously acquires imagery and projects these images onto the assumed ground plane to construct an orthomosaic. At start-up, user set parameters define the extent and spatial resolution of the orthomosaic to be constructed, defining an image whose pixel coordinates can be mapped directly to (x,y,z) positions in the common coordinate frame. As each image is acquired, the estimate of the pose of the camera relative to this ground plane image is used to compute a homography between the two. Using this homography, color values for the pixels of the ground plane image can be drawn from the captured images in real time. This process occurs at just over 1 Hz. The current implementation could be sped up by substituting the central processing unit (CPU)-based *warpPerspective* function in OpenCV [23] with the graphical processing unit (GPU) version to leverage the onboard GPU.

Because the homography is computed directly from the UAV’s state estimate, the accuracy of the assembled orthomosaic is determined by the accuracy of its state estimate as well as the accuracy of the time stamps assigned to the captured images. An RTK GPS system provides position observations which are fused into an EKF to reduce the error in the estimated position of the UAV. The UAV’s orientation estimate is computed from integrated rate gyros and is supplemented by an external compass.

The current terrain classifier is a simplified, pixel by pixel, HSV classifier that classifies the assembled image of the ground plane into four classes: asphalt, grass, obstacle, and unknown. The unknown class explicitly refers to areas of the ground plane that have not yet been observed. Asphalt is recognized as as regions with saturation below a user-defined value which handles both the black surface as well as painted white markings on the runway in the test area. An explicit colored obstacle class is detected as a single solid color with a hue within a defined range, and saturation and value above the minimum values. The remaining area is assigned to the grass class. This classifier used hand-tuned thresholds and was not particularly robust to variability in the conditions, it only serves as a placeholder for more sophisticated terrain classification algorithms, such as the algorithm described in [1], which will be integrated at a later date.

Once classes are assigned to each pixel, this class image is then converted to a cost map. Obstacles are denoted with infinite cost, and unknown areas are denoted with a special value. Asphalt and grass are given cost values matching their ratio in [1] at the Jackal’s nominal speed. The cost values are 16 for asphalt and 27 for grass, corresponding to their traversal costs with the Turtle of 0.332 kW for asphalt and 0.543 kW for grass. Once this cost map has been generated, a dilation step must take place to account for the radius of the ground vehicle during planning.

After the UAV has successfully planned and executed each trajectory in the sequence of waypoints, it calls a D* [24] planner running onboard the UAV. This planner retrieves the current ground cost map and plans a path from the UGV’s current position to the UGV goal. As a default, this UGV goal defaults to the last point in the sequence of waypoints specified by the user, but the user may also explicitly specify a goal. For efficiency, the classification and cost map generation step described above only happens at the demand of the planner, rather than continuously. The planned path is then transmitted back to the OCS.

By running image assembly, terrain classification, cost map generation, and global path planning all onboard the UAV, the required bandwidth between the UAV, ground station, and UGV is dramatically reduced. Command waypoints, the planned path, and the current UGV state are the only things that must be transmitted to the aerial vehicle, and this requires very little bandwidth compared to sending imagery and cost maps. However, it is important to note that because obstacle maps are not directly synchronized between the aerial vehicle and ground vehicle, there is opportunity for the two planners to generate different plans when they do not detect the same set of obstacles in an environment. This entire half of the mission is managed by a state machine onboard the UAV which uses action and service interfaces for planning, trajectory execution, and data retrieval which improve reliability and correctly handle potential failures in each of the subtasks. The current state of the execution is relayed back to the operator for debugging purposes.

This planned path for the ground vehicle is visualized on the OCS and if the operator is satisfied with the trajectory, he may send it to the UGV. The UGV has a much simpler state machine which reacts to this incoming path and feeds it into Move Base [15]. Move Base is a 2D navigation stack for ground robots that integrates obstacle mapping, global path planning, local path planning for obstacle avoidance, control, and trajectory execution. A custom global path planning plugin receives the path planned by the UAV, repairs the plan to ensure it is still feasible, and passes it to the rest of the Move Base stack.

The global path plan was generated by planning on terrain based cost map transformed derived from data transformed into the common coordinate frame. That data was generated by sensors attached to a separate robot from the one executing it. This introduces another opportunity for pose estimation error to enter the system. The RTK system equipped to the Jackal keeps its position error down to a few centimeters, but ultimately during execution, the position and orientation error of the UAV, as well as the position error of the UGV within the common coordinate frame will appear as an error between where the UAV thought obstacles were relative to the UGV and where they actually were relative to the UGV. If these errors are large enough, the planned path may pass through an obstacle. There is also the possibility that an obstacle is undetected from the air. Move Base performs obstacle detection using a LIDAR onboard the Jackal and and uses local trajectory planning in an odometry frame to avoid collisions despite these issues. Path following terminates when the vehicle arrives at the destination or if the vehicle is unable to reach the goal.

### 5.4. Implementation Details

In a system this complex, there are quite a few implementation details that often go unmentioned in academic papers. However, to be useful to other members of the community who might wish to construct a similar system, it is important to mention some of the low level details and design choices made in its construction. This section will touch on a few issues common to all multi-robot systems, time synchronization between computers and communication in multi-robot systems, and issues more specific to ROS-based systems.

#### 5.4.1. Time Sync

Chrony [25] is a an implementation of the Network Time Protocol (NTP) used to synchronize the system clocks of the onboard computers with the system clock of the OCS. ROS utilizes time stamps derived from the current system time on each computer whenever a node looks up what the current time is. For example, the TF [26] package in the ROS maintains a transform tree which utilizes the Universal Robot Description Files (URDF) in conjunction with sensor data to track the transformations between different frames defined for the system as they change with time. Any node may add to this transform tree by publishing transforms between frames at particular times. This system inherently assumes that there is a single consistent source of time for the ROS system, so when nodes are running on multiple computers, or in this case on multiple separate robots, it is necessary to synchronize the system clocks between the computers to avoid violating this assumption.

#### 5.4.2. Comms Bridge

The ROS is a flexible middleware employed in a wide range of robots, but it has some shortcomings in multi-robot systems containing unreliable wireless links. This system implements a custom ROS to ROS bridge which manages bandwidth between the robots, reasons over the state of the wireless link, and improves total bandwidth. The main function of this comms bridge is to split the multi-robot system into several separate ROS masters. Each of the two vehicles and the OCS have their own separate ROS master with their own *roscore* running locally. The comms bridge is implemented as a pair of nodes on each side of the two wireless links. These nodes subscribe to ROS topics, process them, and emit messages which are received by the other node in the pair using ZeroMQ, [27], another message-passing library.

Large messages, such as paths, are broken up into smaller chunks and then re-serialized using a flexible combination of ROS serialization and Message Pack [28]. Conversely, small serialized messages are packed together to hit a 1500 byte payload target. This payload target maximizes throughput by minimizing the impact of dropped packets on bandwidth. The comms bridge utilizes the User Datagram Protocol (UDP), rather than Transmission Control Protocol (TCP) to transmit between robots which further minimizes overhead. The comms bridge itself handles retransmission at a higher level in ways that are specific to each type of data being sent. For example, when transmitting cost maps across the comms bridge, the two halves of the bridge use a synchronization scheme where the map is broken up into individual tiles. The downstream side has a copy of the map and requests updates from the upstream side. If a tile is dropped in transmission, then the downstream side remains out of date and will request the tile again. Because the upstream side always sends the most recent copy of a tile, it avoids transmitting the unnecessary intermediate states of the tile.

Importantly, from a safety perspective, each side of the comms bridge tracks when the last message was received from the other side. If messages are not being received, then the connection has been lost and both robots will automatically cancel their actions and stop their motions.

#### 5.4.3. UAV State Transitions

On hardware, for safety reasons, the autonomous system does not broadcast commands to transition the flight controller’s state into the software-controlled *GUIDED* state. The pilot in command is prompted to change flight modes manually while the system waits to confirm the transition before continuing with trajectory execution. The system switches back to *LOITER* on the completion of a trajectory to ensure that the aircraft is in a safe state. While this approach is more tedious for the pilot, it does give them final authority over the movement of the vehicle and avoids the possibility of any unexpected state transitions being caused by the autonomous system while it is under manual control.

#### 5.4.4. TF Tree and Namespaces

To ease the creation and debugging this software system, it was laid out in a way that keeps the launch files virtually identical between simulation and hardware with both single and separate ROS masters.

Within a single ROS master, all nodes must have unique names. The ROS encourages the use of namespaces to organize nodes. Placing a node into a namespace causes relative topics published and subscribed to by that node to be prepended with the namespace. By launching all nodes within a robot specific namespace regardless of whether they are being run in simulation or hardware, all of the node names and topic names will remain consistent. As long as each robot is given a unique name, multiple copies of the same robot can be run from the same launch file.

**Figure 6:**
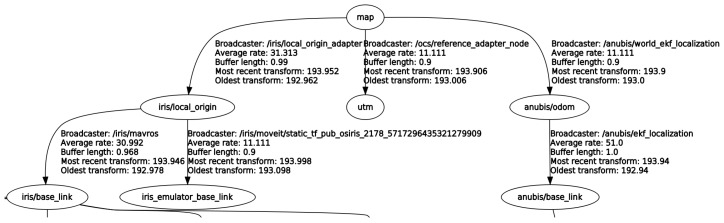
Excerpt of TF tree of the system in simulation. Both vehicles have a $base_{l}ink$ frame. Since they are prepended by the vehicle names “iris” and “anubis”, the frames are unique.

Link names within the TF tree must also be uniquely named. The transform tree is a somewhat unique in the ROS in that all nodes that interact with the TF tree publish and subscribe to the same *\tf* topic. This means that if a single ROS master is used across multiple identical robots, there is the issue of ensuring that all of the link and joint names remain unique. One possibility would be to maintain multiple separate URDFs for each robot, but this would be redundant. Instead, this system uses a xacro [29] version of the URDF which allows a robot name argument to be prepended to each link and joint name in the URDF. Figure 6 shows an excerpt of the TF tree demonstrating this idea. However, this requires all parts of the system to conform to the convention, which requires modification to the interface software used for the Jackal as well as the implementation of an adapter to modify the names of links referred to in topics being published by an ROS serial interface from a micro-controller built into the Jackal.

## 6. Experiments

Preliminary experiments to investigate the effects of field of view on the UAV’s camera on the quality of planned paths for the UGV were initially conducted in Matrix Laboratory (MATLAB). In these trials, the area imaged by the UAV while moving to the goal was simulated by extracting subsets of a pre-collected orthomosaic and the size of this area was varied. Then, a path planner was run on the classified terrains to examine the change in path cost as additional area was made available for planning.

These were followed by experiments in both a simulated Gazebo environment and at Kentland Farm, a Virginia Tech-operated farm that allows the testing of UAVs.

In simulation, a flat ground plane was created and colored obstacles were added. The ground plane was textured with an orthomosaic captured from Kentland Farm and assembled using Agisoft PhotoScan [30] to enable the quality of the image assembly method to be qualitatively evaluated. This simulation environment enabled the majority of the software created for the system to be tested including the planning, navigation, and obstacle detection for both vehicles.

When running on a single computer with a single ROS master, the comms bridge is also being tested. However since messages are passed over the loop back interface, it has much lower latency and higher bandwidth than on hardware. Simulation testing of multiple separate ROS masters on separate computers was conducted to validate the comms bridge before deploying to hardware with the wireless link in the loop.

**Figure 7:**
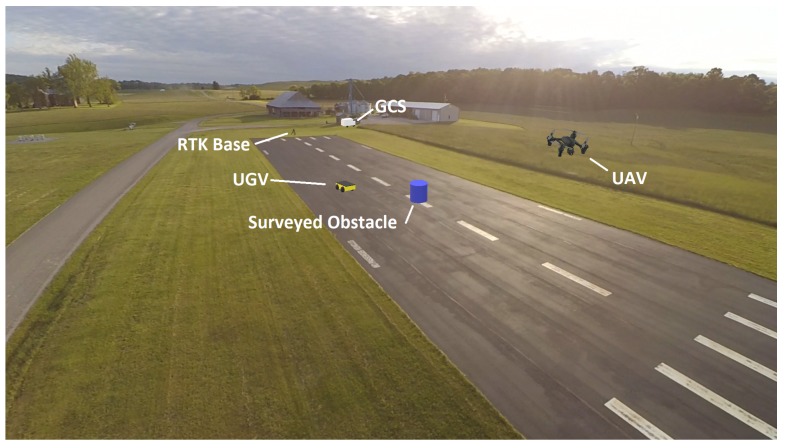
Kentland Farm experimental setup. GCS: Ground Control Station.

During initial testing and debugging on hardware, the UAV was not flown, but its software was kept in the loop by substituting the terrain-based cost map with the obstacle map generated by Move Base. For full system testing, a flat area in the vicinity of a small test runway at Kentland Farm was used for several experiments, as shown in Figure 7. End to end system tests were conducted, where the UAV was launched and an operator validated the behavior of the complete system. The imagery assembled by the aerial vehicle was qualitatively evaluated, revealing some issues with the current algorithm.

A color-coded blue trash can was placed on the runway as an obstacle. The position of this obstacle was surveyed with an additional RTK unit to compare its true location with the locations estimated by both the UAV and UGV. After the full system test where it was confirmed that the UAV identified the obstacle from the air and planned a path around it, and it was confirmed that the UGV successfully detected the obstacle and avoided it while executing the path planned by the UAV, an additional test was conducted to for explicit examination of the position errors in the system.

## 7. Results

**Figure 8:**
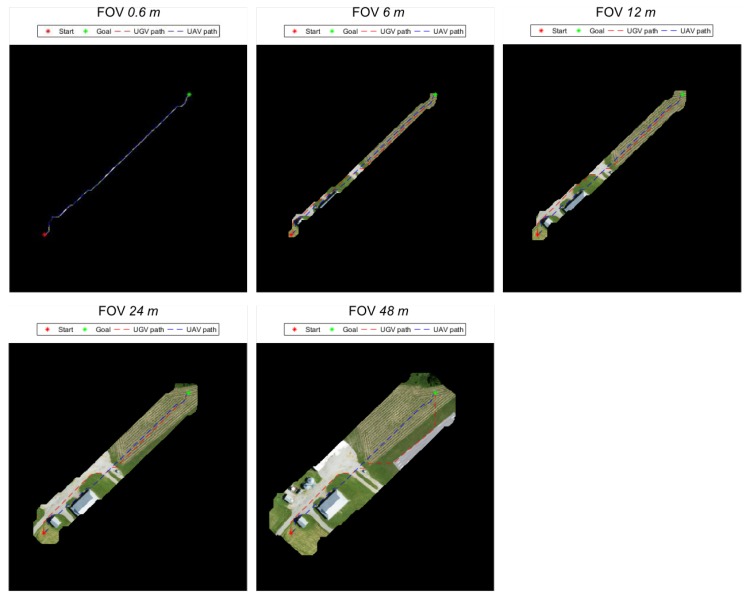
Paths corresponding to cases presented in Table 1. As the field of view of the simulated camera is expanded, the path cost is reduced despite an increase in distance traveled.

Figure 8 shows a graphical representation of the effects of varying the effective field of view of the aerial vehicle on the planned paths for the ground vehicle, while Table 1 shows the quantitative effects. As expected, a larger field of view allows the path planner for the ground robot to generate lower cost paths by reducing the distance driven over the more expensive grass class, even if it comes at the cost of a longer overall plan. When the Field of View (FOV) increases from 24 m to 48 m, we see a marked change in the planned path which detours over to the runway. In this same way, we also expect improvements in distance traveled when faced with obstacles rather than just more costly terrain. In more obstacle-dense environments, the aerial vehicle’s perspective has the additional benefit of not being blocked. This should allow dead-ends to be more quickly identified from the air and result in less doubling back than would be required of the ground vehicle alone.

**Figure 9:**
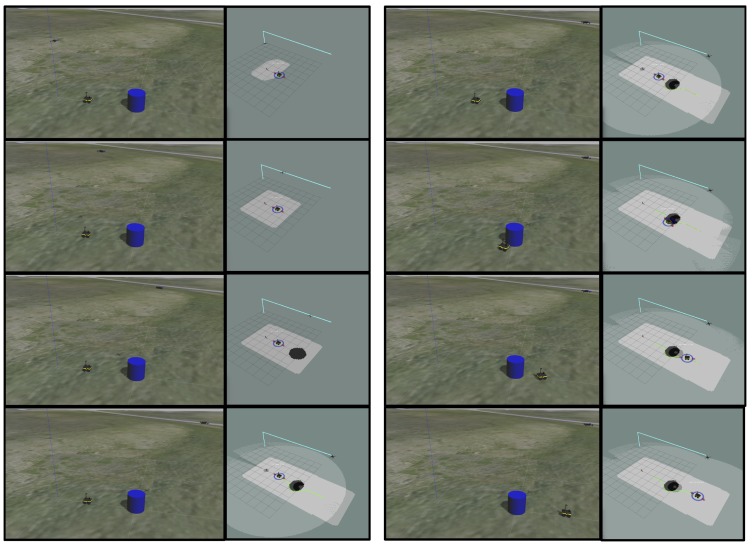
This sequence of images, descending down the left column and then the right column, shows a brief test in simulation. The left window in each pair shows the Gazebo environment, and the right window shows the RViz window. From fourth image on, the ground-based obstacle map is also visualized.

Figure 9 shows a typical execution of the mission in simulation. The user clicks on a point on the right hand side of the environment to specify a goal for the system. The UAV generates a path, shown in light blue, ascending to the desired altitude and flies to the destination. The expanding rectangular area of light grey in the right-hand RViz window is the expanding area of observed and classified terrain in the terrain map. The black circle that appears in the terrain map is the detected blue obstacle. Once it has arrived at the end of the path, a trajectory for the ground vehicle, shown in green, is planned. The UGV then executes the planned path avoiding the obstacle by following the plan and using its onboard LIDAR. The light grey circular region in the right hand RViz window is the obstacle map generated by the onboard LIDAR. This onboard obstacle detection enables the ground vehicle to avoid the obstacle despite the parallax error between the aerial constructed terrain map and the ground-level obstacle map.

**Figure 10:**
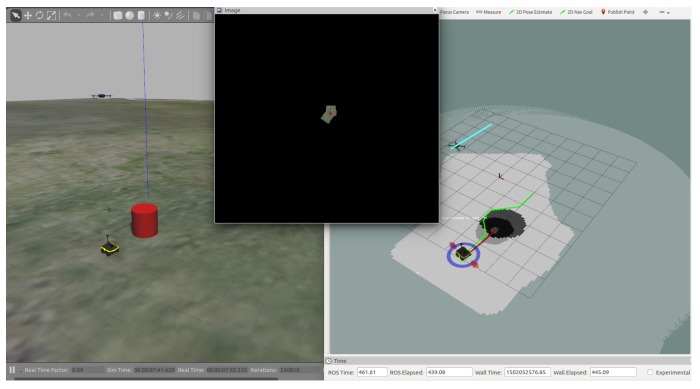
This simulation illustrates the issue of parallax error in the estimated obstacle location. The left half of the figure displays the Gazebo simulation environment, featuring a ground vehicle, an aerial vehicle, and the obstacle. The right half of the figure displays two obstacle maps overlaid on one another. The ring shaped region shows the perimeter of the object as seen by the ground vehicle, while the solid, larger circle displays the obstacle as detected by the aerial vehicle. Note the offset between them as well as the difference in estimated size which is due to the position of the aircraft directly above the ground vehicle and offset from the center of the obstacle.

Figure 10 more clearly illustrates the parallax error inherent in the 2D assumption of the ground plane reconstruction. The parallax error is an error in the estimated position of an obstacle from the air if the height of the UAV is small compared to the height of the obstacle.

Hardware testing confirmed that the above mission functioned on hardware with a few caveats. With respect to the hardware, take off and landings are performed manually by the pilot in command. In simulation, the system autonomously makes state transitions on the flight controller automatically. On hardware, the pilot in command is responsible for making this transition at the request of the autonomous system. This procedure works as expected. However, if this transition is not made in a timely fashion, the trajectory execution of *MoveIt!* will timeout since it expects the trajectory to be completed with a certain period of time.

There is an issue of operator immersion in the OCS which makes it difficult to specify goals in unexplored areas. This lack of presence could be addressed by incorporating coarse pre-collected imagery of the area into the visualization similar to other ground station applications, such as Mission Planner.

Overall, the system did function as intended on hardware. The operator was able to specify goals for both vehicles and move them to the specified locations. The online image assembler accepted imagery at 1.37 Hz, which was fast enough to avoid gaps in the map when flying at 10 m above the ground surface and traveling horizontally at around 4 m per second.

**Figure 11:**
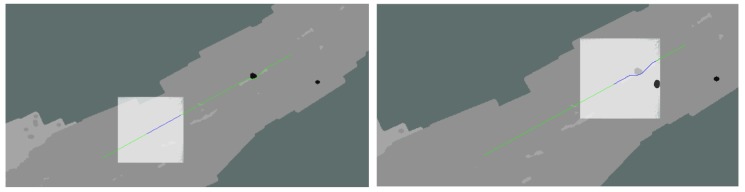
This figure displays the terrain-based cost map, planned ground vehicle path, and local ground-level obstacle map as the vehicle executed the path. The green line shows the planned path of the ground vehicle, while the blue shows the subset of this planned currently being followed. Note the false positive obstacle detected to the right of the planned path. The white square shows the overlaid ground-level obstacle map. Note in the right-hand image, the black circle at the edge of the local cost map does not align with the obstacle as seen from the air.

The ground vehicle successfully avoided the obstacle in each test, an example of which is shown in Figure 11, however, the error between the aerial constructed terrain map and the ground-level obstacle map was great enough that obstacle avoidance was due to the UGV’s onboard replanning, and in the case noted above, the path simply missed the obstacle entirely.

Two tests to characterize the absolute and relative errors in the estimated locations of the obstacle from the aerial vehicle and from the ground vehicle were conducted. In a more realistic scenario, the UAV was manually flown over the obstacle to gather imagery, and a plan was constructed, sent to the UGV, and then executed. The location of the obstacle in the aerial terrain map and the ground-level obstacle maps were extracted as the centroid of the obstacle cells within the two maps and compared to the ground truth position of the obstacle obtained by surveying its position with an RTK GPS. Table 2 shows the results of these trials. The test was broken up into two flights due to limitations in battery life, but the obstacle was not moved between the first six trials and the second six. Since planning for the UAV is done after the UAV has completely flown past the obstacle, the final estimated position of the obstacle was used. It was observed that while the UGV was driving past the obstacle, the estimated location of the obstacle in the map frame shifted quite substantially. There are two factors at work. The first is that the UGV only observes one side of the obstacle with its LIDAR. Since it does not observe the entire perimeter of the obstacle at once, the centroid of the obstacle pixels in the obstacle map will be the center of this observed arc, rather than the center of the circle that it represents. The second and more dominant source of error is due to error in the estimated orientation of the ground vehicle. This causes the position of the obstacle to shift substantially as the ground vehicle approached the obstacle. To try and quantify this error, both the average and the maximum errors are listed. Because obstacles are only inserted up to 5 m away, the errors introduced by this error in orientation are bounded.

One other issue that appeared when taking repeated passes at the obstacle with the UAV was the duplication of the obstacle in the terrain map before the UAV had completed its flyover. If the obstacle is on the edge of the captured image, and there is enough error in the state estimator that the projection of the image does not overlap with the previously projected image of obstacle in the map, it is possible to create duplicate copies of the obstacle in the assembled image.

**Figure 12:**
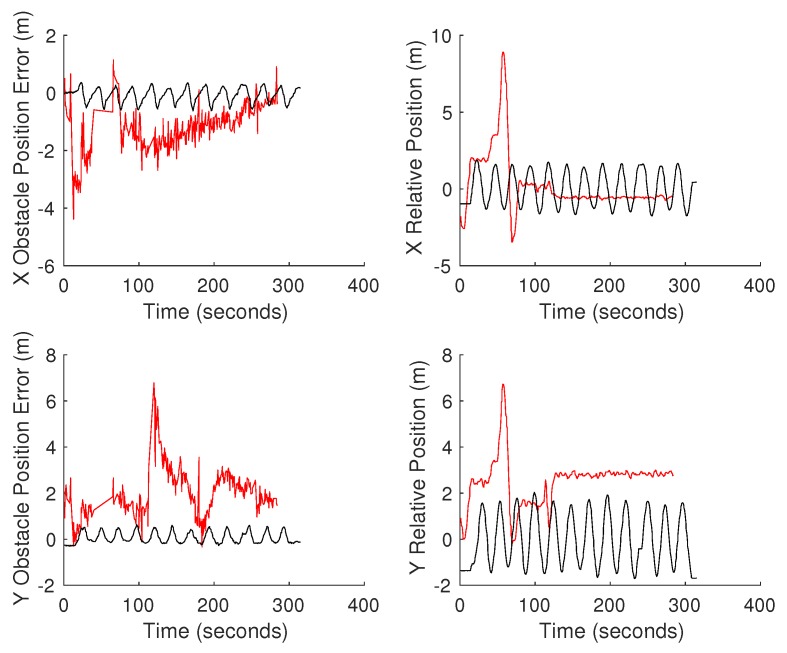
Continuous obstacle position test conducted with first-generation Piksi. Red shows the data associated with the UAV, while black is the data for the UGV.

**Figure 13:**
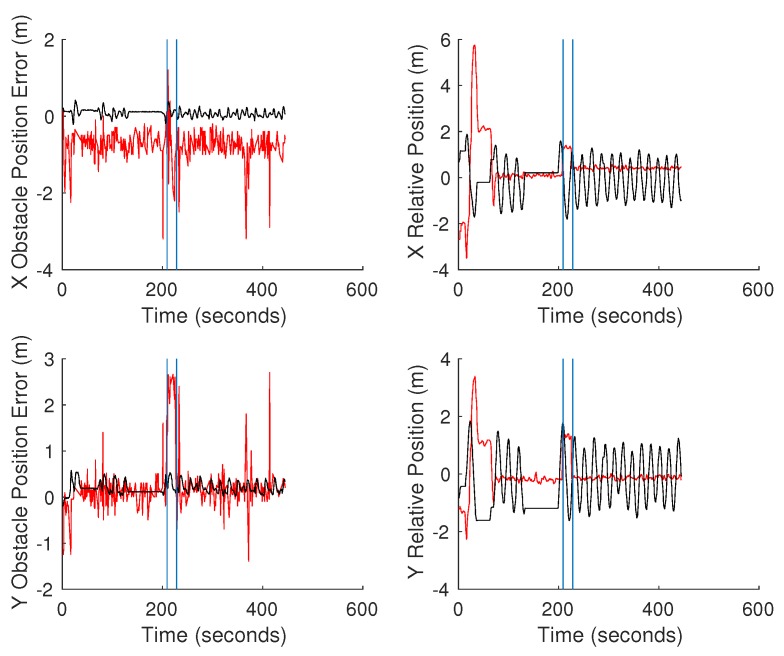
Continuous obstacle position test conducted with the Piksi Multi. Red shows the data associated with the UAV, while black is the data for the UGV. The vertical blue lines indicate the period of time where the RTK GPS on the UAV failed.

The second test to quantify the errors between the estimated obstacle locations was to hover the UAV over the obstacle and manually drive the ground vehicle in circles around the obstacle. The ground vehicle was driven around the obstacle to average out the effect of only viewing a portion of the obstacle at a time. Two separate tests were conducted. The first test, shown in Figure 12, was conducted with the first-generation Piksi RTK GPS. We notice the oscillation in estimated position of the obstacle from the UGV related to the path driven by the vehicle. This oscillation was caused by an offset in the orientation of the vehicle and its true orientation due to an issue with the application of magnetic declination correction. This issue was corrected in the second test shown in Figure 13. In the second test, the vehicles were also switched over to the Piksi Multi RTK GPS. This second test shows significantly improved errors except for an unexplained consistent offset in the X position of the location of the obstacle estimated by the UAV. Table 3 more clearly illustrates the improvement in error when the compass error was corrected on the UGV and the general improvement to the system when a more robust RTK GPS was used.

**Figure 14:**
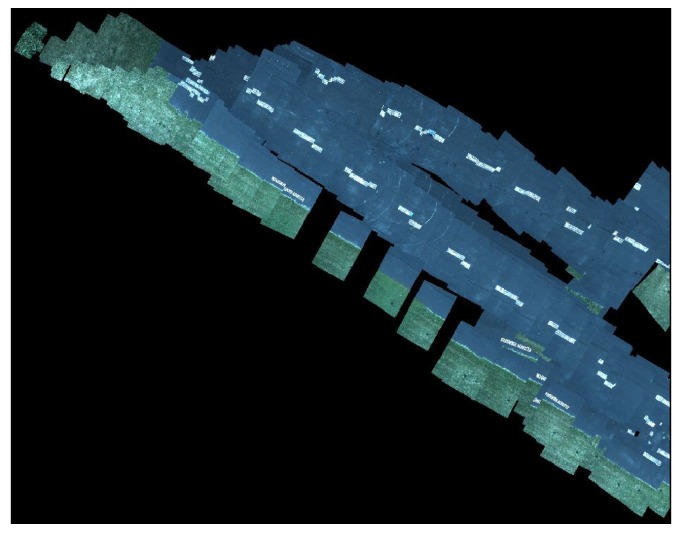
Discontinuity in assembled aerial imagery due to switching from single point position (SPP) fix to RTK fix. *Note that the runway only has a single center white dashed line.*

**Figure 15:**
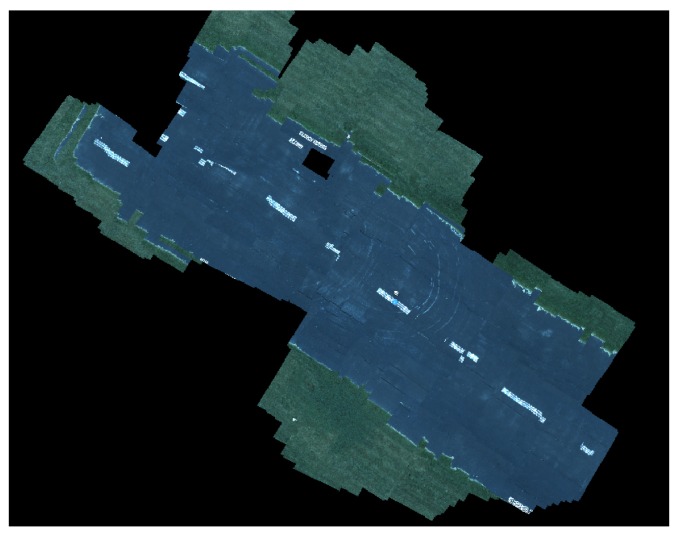
Successful assembly of image of runway showing a more normal result. *Note this figure is not at the same scale as Figure 14.*

One of the biggest failure modes encountered during hardware testing was the RTK GPS onboard one of the vehicles losing its RTK fix. In early testing, this was usually an issue with the UAV which has several other radios and more unshielded electronics, which interfered with the correction radio. The Swifnav RTK units do not send out RTK fix positions unless it has recently received an RTK correction message from base station. The Pixhawk expects GPS updates at a regular rate, and in the event that the RTK GPS misses an update deadline, it will switch over to a backup GPS receiver onboard. However, because this other GPS receiver position is not augmented, it has slowly time-varying errors in position that appear as an offset between the position reported by the augmented and un-augmented fixes. When the UAV switches GPS units, its position estimate jumps, most obviously appearing as a discontinuity in the assembled imagery as shown in Figure 14. Figure 15 shows a typical result, even with a stable RTK fix, the orientation estimate errors are still visible as small discontinuities.

Despite these large errors between both the estimated obstacle positions and the true position, as the ground vehicle never collided with the obstacle, its onboard obstacle avoidance was sufficient. However, this level of error is great enough that paths taken by the ground vehicle might not remain in the correct class. For example, executing a path planned down the side of a road with 1 m of error might mean that the ground vehicle drives on the grass to the side of the road instead of on the road itself. This issue could be mitigated by dilating the cost map generated by the aerial vehicle by an amount related to this error, but it would be preferable to reduce the positioning error.

## 8. Future Work

One of the main goals of future work will be to remove the planar environment assumption from the aerial vehicle’s mapping. Using a stereo camera pair on the UAV, rather than a monocular camera, will allow the system to construct height maps of the environment using the depth image. This will eliminate parallax errors by explicitly modeling terrain height and allow obstacles to be detected as discontinuities in height rather than an arbitrary color. This transition will also require the use of feature point tracking to aid in the reconstruction by providing better estimates of the change in position and orientation from one pair of images to the next. Depending on the quality of the reconstruction that can still be achieved in real time, it may also be necessary to implement some smoothing technique to avoid introducing height discontinuities at the edges of the observed depth image due to slight misalignments.

In future work we would also like to more tightly close the loop between the planning and motion of the UAV and UGV.

The current planner for the UAV flies between a sequence of waypoints specified by the user, as a simplified method to collect imagery of the area we expect the ground vehicle to drive through. However, in an environment with a wall or other extended obstacle, this does not guarantee that the aerial imagery will contain a feasible path for the UGV. Instead, the planner and trajectory execution of the UAV should wrap around the ground vehicle planner so that it continues to search the area until a path for the ground vehicle is discovered.

The system should also somehow synchronize obstacle and terrain cost data between the two vehicles. When the UAV plans a path for the ground vehicle, it does not have knowledge of the obstacles that the ground vehicle has discovered. If the ground vehicle observes a large wall that does not appear in the aerial obstacle map, then the ground vehicle will identify that the aerial vehicle’s planned path is infeasible because it might pass through an obstacle. The UGV can replan a completely new path that avoids this obstacle to travel to the goal, but because it is planning without the terrain-based cost map on the UAV, its path will no longer minimize energy consumption. Synchronizing the data between the two robots will lead to more consistent planning behavior in challenging environments.

We also wish to use the path execution by the ground vehicle as feedback for the terrain classification. Under a reinforcement learning scheme, the classes of terrain could be corrected as the ground vehicle drives on them. For example, a dark gravel that was mistakenly classified as asphalt might be correctly identified by the higher energy cost to drive over it.

In another scheme, this feedback could be used to update energy costs associated with different terrains. In the current system, the ground vehicle planner utilizes terrain costs estimated offline from previously collected data. However, a wide variety of circumstances, such as recent weather turning dirt to mud, could alter terrain costs substantially from their values in the training set. This could be partly addressed by expanding the terrain classifier to cover more unique classes, distinguishing between dirt, mud, swap, etc. However, another way to address the shortcoming of the original training data is to revise terrain costs as the vehicle traverses it. Without having to split dirt into a separate mud and dry dirt classes, observing the energy cost of driving over mud, so long as the vehicle can do so safely, would let the system adjust the dirt class’s cost upwards. Replanning with the new cost will produce a good result without introducing more granularity into the classifier.

## Figures and Tables

**Table 1:** The UGV planned path energy cost vs. aerial imagery field of view. Note that while total path length increases, energy cost decreases because of the reduced distance driven on grass. The paths are shown in Figure 8.

Trial	Field of View (m)	UGV Path Length (m)	UGV Traversal Cost (kJ)	Terrain Type	
				Asphalt (m)	Grass (m)
1	0.6	229.83	58.51	36.85	192.99
2	6	234.51	57.00	63.22	171.29
3	12	235.01	54.59	87.33	147.68
4	24	235.21	52.54	107.25	127.96
5	48	255.04	51.21	170.96	84.09

**Table 2:** Results from passes made by both vehicles at the obstacle. The absolute errors refer to the distance between each vehicles’ estimate of the position of the obstacle and the true real-time kinematic global positioning system position. The relative error is a comparison between the estimated positions from each vehicle.

Test	Absolute UAV Error (m)	UGV Average Absolute Error (m)	UGV Max Absolute Error (m)	Average Relative Error (m)	Max Relative Error (m)
1	2.148	0.822	1.898	1.857	2.947
2	0.371	0.242	0.447	0.375	0.628
3	1.819	0.810	1.533	1.818	2.740
4	1.017	0.748	1.597	1.064	2.414
5	1.518	0.395	0.962	1.519	1.829
6	1.583	0.570	1.208	1.714	1.984
7	1.009	0.789	1.721	1.054	2.256
8	1.011	0.465	0.690	1.291	1.475
9	0.776	0.781	1.511	1.296	2.251
10	0.923	0.374	0.729	1.111	1.328
11	0.648	0.869	1.635	1.241	1.618
12	1.169	0.685	1.540	1.411	1.732

**Table 3:** Average and standard deviations of errors in hover tests shown in Figure 12 and Figure 13. The results for the second test exclude the data from the UAV during the period of RTK failure.

Hover Test	Average UAV Absolute Error (m)	Standard Deviation UAV Absolute Error (m)	Average UGV Absolute Error (m)	Standard Deviation UGV Absolute Error (m)
1	2.501	0.907	0.330	0.164
2	0.814	0.432	0.236	0.122

## Abbreviations

The following abbreviations are used in this manuscript:

LIDAR	Light detection and ranging
UAV	Unmanned aerial vehicle
UGV	Unmanned ground vehicle
GPS	Global positioning system
RTK	Real-time kinematic
IMU	Inertial measurement unit
INS	Inertial measurement system
OCS	Operator control station
ICP	Iterative closest point
ROS	Robot operating system
RC	Radio Control
DOF	Degree of freedom
CPU	Central processing unit
GPU	Graphical processing unit
IP	Internet protocol
EKF	Extended Kalman filter
HSV	Hue Saturation Value
SIFT	Scale-Invariant Feature Transform
NTP	Network Time Protocol
UDP	User Datagram Protocol
GCS	Ground Control Station
MATLAB	Matrix Laboratory
FOV	Field of View
TCP	Transmission Control Protocol
